# Reviewer acknowledgement 2015

**DOI:** 10.1186/s40942-016-0032-0

**Published:** 2016-02-16

**Authors:** Jay Duker, Eduardo Rodrigues

**Affiliations:** 1 Tufts University School of Medicine, Boston, USA; 2 Federal University of São Paulo, São Paulo, Brazil

## Abstract

The Editors of *International Journal of Retina and Vitreous* would like to thank all of our reviewers, both external and Editorial Board Members, who have contributed to the journal in Volume 1 (2015) and whose valuable support is fundamental to the success of the journal.

**Acácio Muralha**

Brazil

**Aderbal de Albuquerque Alves**

Brazil

**Airton Kronbauer**

Brazil

**Alessandro Daré**

Brazil

**Alex Hunyor**

Australia

**Alexandre Ventura**

Brazil

**Alice Degoumois**

Brazil

**Anat Loewenstein**

Israel

**Andre Gomes**

Brazil

**Andre Romano**

Brazil

**Antonio Marcelo Barbante Casella**

Brazil

**Arnaldo Furman Bordon**

Brazil

**Arturo Alezzandrini**

Argentina

**Baruch Kuppermann**

United States of America

**Caio Regatieri**

United States of America

**Caio Augusto Scocco**

Brazil

**Carl Regillo**

United States of America

**Carlos Veloso**

Brazil

**Carol Shields**

United States of America

**Carsten Meyer**

switzerland

**Chen Hao-Yu**

China

**Christopher Brady**

United States of America

**Daniel Lavinsky**

Brazil

**Daniela Ferrara**

United States of America

**David Boyer**

United States of America

**David Leonardo Isaac**

Brazil

**Debra Goldstein**

United States of America

**Eduardo Souza**

Brazil

**Eduardo Novais**

Brazil

**Eduardo Cunha de Souza**

Brazil

**Emmerson Badaró**

Brazil

**Enzo Fulco**

Brazil

**Evandro Lucena**

Brazil

**Fang Wang**

China

**Fernando Penha**

Brazil

**Fernando Arevalo**

Saudi Arabia

**Fernando Jose de Novelli**

Brazil

**Focke Ziemsen**

Germany

**Francesco Rodriguez**

Colombia

**Francisco Max D’Amico**

Brazil

**Gregory Lee**

United States of America

**Helio Francisco Shiroman**

Brazil

**Hidenori Takahashi**

Japan

**Irena Tsui**

United States of America

**Jaime Roizenblatt**

Brazil

**James Augsburger**

United States of America

**Jay Chhablani**

India

**João Dias**

Brazil

**João Luiz Lobo Ferreira**

Brazil

**John Helal Junior**

Brazil

**Jorge Mitre**

Brazil

**Jorge Carlos Pessoa Rocha**

Brazil

**Jose PUlido**

United States of America

**Junior Kuczmainski**

Brazil

**Kleyton Barella**

Brazil

**Kouros Panagiotis**

Switzerland

**Koushik Tripathy**

Brazil

**Laurentino Biccas**

Brazil

**Leandro Cabral Zacharias**

Brazil

**Leonardo Cunha Castro**

Brazil

**Lihteh Wu**

Costa Rica

**Luiz Lima**

Brazil

**Luiz Henrique Lima**

Brazil

**M Chéour**

France

**Magno Ferreira**

Brazil

**Makoto Inoue**

Japan

**Marcin Stopa**

Poland

**Marcio Nehemy**

Brazil

**Mariana Matioli**

Brazil

**Mário Nóbrega**

Brazil

**Mary E Hartnett**

United States of America

**Mathew MacCumber**

United States of America

**Matus Rehak**

Germany

**Mauricio Maia**

Brazil

**Mauro Goldbaum**

Brazil

**Michael Singer**

United States of America

**Michel Farah**

Brazil

**Minoru Furuta**

Japan

**Muhammad Moazam Fraz**

Pakistan

**Murat Hasanreisoglu**

Turkey

**Nicolas Feltgen**

Germany

**Noemi Lois**

United Kingdom

**Ossewaarde-van Norel**

Netherlands

**Paulina Haas**

Germany

**Paulo Augusto de Arruda Mello Filho**

Brazil

**Peter Traine**

Switzerland

**Priscilla Ballalai**

Brazil

**Quan Nguyen**

United States of America

**Rafael Caiado**

Brazil

**Raja Narayanan**

India

**Rajendra Apte**

United States of America

**Ranjana Mathur**

Singapore

**Raul Vianna**

Brazil

**Rodrigo Meirelles**

Brazil

**Rodrigo Lira**

Brazil

**Sandeep Saxena**

India

**Serdar Bilici**

Turkey

**Sérgio Pimentel**

Brazil

**Szilard Kiss**

United States of America

**Taiichi Hikichi**

Japan

**Tatsuhiko Sato**

Japan

**Thomas Theelen**

Netherlands

**Vincent Daien**

France

**Vinicius Kniggendorf**

Brazil

**Vinícius Saraiva**

Brazil

**Zihret Abazi**

Serbia and Montenegro

